# Attempted aspiration of a symptomatic lumbar juxtafacet cyst resulting in improvement of acute onset radiculopathy with progressive motor weakness

**DOI:** 10.1016/j.inpm.2022.100101

**Published:** 2022-05-30

**Authors:** Sharlene Su, Yumi Mitsuya, Derek Schirmer, Josh Levin

**Affiliations:** a Department of Orthopedic Surgery, Stanford University, USA; b University of California San Francisco Benioff Children's Hospitals, USA; c Department of Neurosurgery, Stanford University, USA

Dear Editor,

Facet joint cysts are often found in the presence of degenerative changes and are most frequently found at the L4-5 level [1,2]. When large enough, facet joint cysts can cause radicular pain, neurogenic claudication, sensory loss, and motor weakness (27%)[3]. Cauda equina syndrome also has been reported[4]. Percutaneous treatment options include cyst aspiration, cyst rupture, and intra-articular facet joint steroid injection. Traditional surgical management consists of laminectomy with cyst resection with or without spinal fusion.

Several studies have evaluated outcomes of percutaneous facet joint cyst rupture in combination with intra-articular facet joint steroid injection. A retrospective study of 101 subjects who received an intra-articular facet joint steroid injection followed by an attempted facet joint cyst rupture (cyst rupture was successful 81% of the time) showed that 46% had symptom relief and avoided surgical treatment [5]. In another retrospective cohort of 32 patients who underwent fluoroscopically guided cyst rupture followed by intra-articular facet joint steroid injection, 72% achieved excellent long-term pain relief and 81% avoided surgery [6]. Another retrospective study of 110 subjects who underwent attempted facet joint cyst rupture (cyst rupture successful 87% of the time) followed by intra-articular facet joint steroid injection showed that 60% averted surgical intervention, while 71% of those with high cyst T2 signal intensity were able to avoid surgery at a mean of 34 months after percutaneous rupture [7]. In a recent prospective study of 35 subjects, percutaneous facet joint cyst rupture (cyst rupture successful 94% of the time) followed by intra-articular facet joint steroid injection yielded clinically and statistically significant pain relief at two and six weeks, though 40% required repeat cyst rupture at a median of 62 days. Overall, 69% were able to avoid surgical treatment [8].

There are fewer reports of direct facet joint cyst aspiration. A recent study evaluated CT-guided intra-articular facet joint steroid injection followed by facet joint cyst aspiration and fenestration. This procedure was performed on 64 subjects (presence or absence of motor weakness was not reported). Fifty-six percent had partial or complete relief at 49 months, while 44% underwent surgical treatment [9].

Lastly, a systematic review and meta-analysis of 870 total subjects with lumbar facet joint cysts showed a 58% rate of symptomatic resolution from percutaneous procedures, and 90% for surgical decompressive procedures. Repeat procedures were required in 29% of percutaneous procedures and less than 1% surgical procedures [10].

Overall, it appears as though approximately half of patients who undergo percutaneous procedures for lumbar facet joint cysts can avoid surgical treatment.

We present an interesting case of a patient with acute onset radiculopathy with a severe and progressive motor deficit from a juxtafacet cyst at L3-4 causing severe canal stenosis. He experienced significant improvement in both pain and weakness after an attempted aspiration, which did not yield any aspirate, but may have resulted in cyst puncture with subsequent slow drainage. This case illustrates that even in the setting of a significant motor deficit, patients presenting with an acute radiculopathy may benefit from percutaneous cyst drainage and be able to avoid surgical intervention.

A 64-year-old previously healthy man presented with three weeks of bilateral leg pain that was exacerbated by standing and walking. On exam, right foot intrinsic and ankle dorsiflexion strength was <3/5 and right knee flexion and hip abduction were 4-/5. He was unable to heel walk on the right side and required a cane for safe ambulation. Left leg strength was 5/5 throughout. He denied any numbness, tingling, or bladder/bowel problems. Lumbar spine MRI revealed a complex cyst arising from the left L3-4 facet joint extending posteriorly/centrally along the ligamentum flavum causing severe canal stenosis (Fig. 1). Given the severe and progressive neurologic deficit, the patient was scheduled to undergo a laminoforaminotomy with decompression of the left L3-4 facet joint cyst to be performed five days later.

**Fig. 1:**
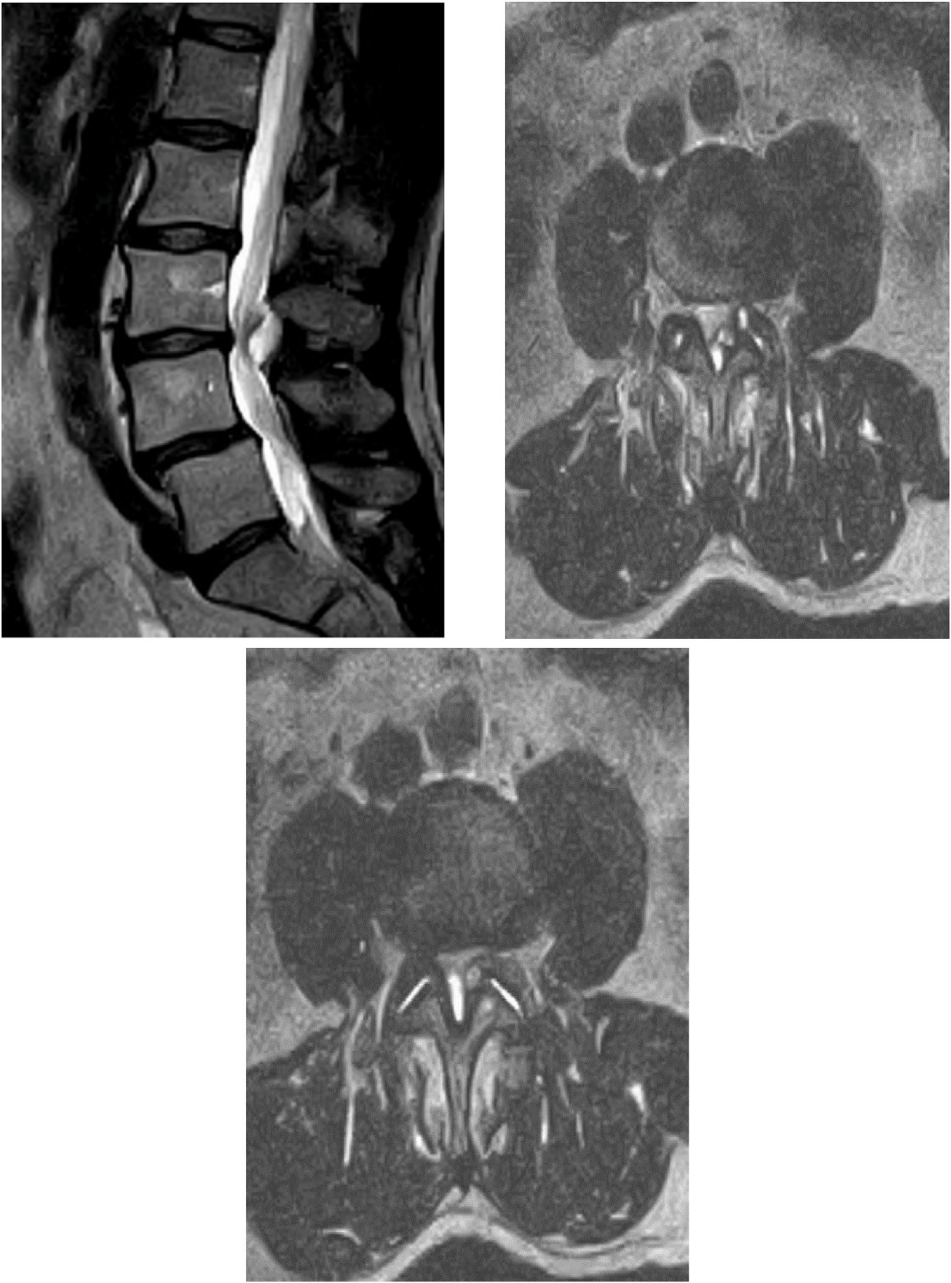
T2 sagittal and axial MRI demonstrating a complex left L3-4 juxtafacet cyst with resultant severe canal stenosis.

While awaiting the operation, at the neurosurgeon's request, the patient underwent an attempted facet joint cyst aspiration through an interlaminar approach, and a left L3-4 intra-articular facet joint steroid injection. The aspiration was first attempted with a 25-gauge spinal needle. After no fluid was able to be aspirated from the cyst, the procedure was re-attempted with a 22-gauge needle. Again, no fluid was able to be aspirated. Another 25-gauge needle was placed within the facet joint and a facet joint arthrogram showed no communication of contrast into the facet joint cyst (Fig. 2). Further ventral needle advancement of the 22-gauge needle that was used for the facet cyst puncture was ceased after the lateral views demonstrated needle placement anterior enough to be within the cyst. Since the facet joint arthrogram did not show a direct communication with the cyst, a total of 0.5 ​cc (40mg methylprednisolone/ml with 1% lidocaine) was then injected into the left L3-L4 facet joint (no medication was injected directly into the cyst).

**Fig. 2:**
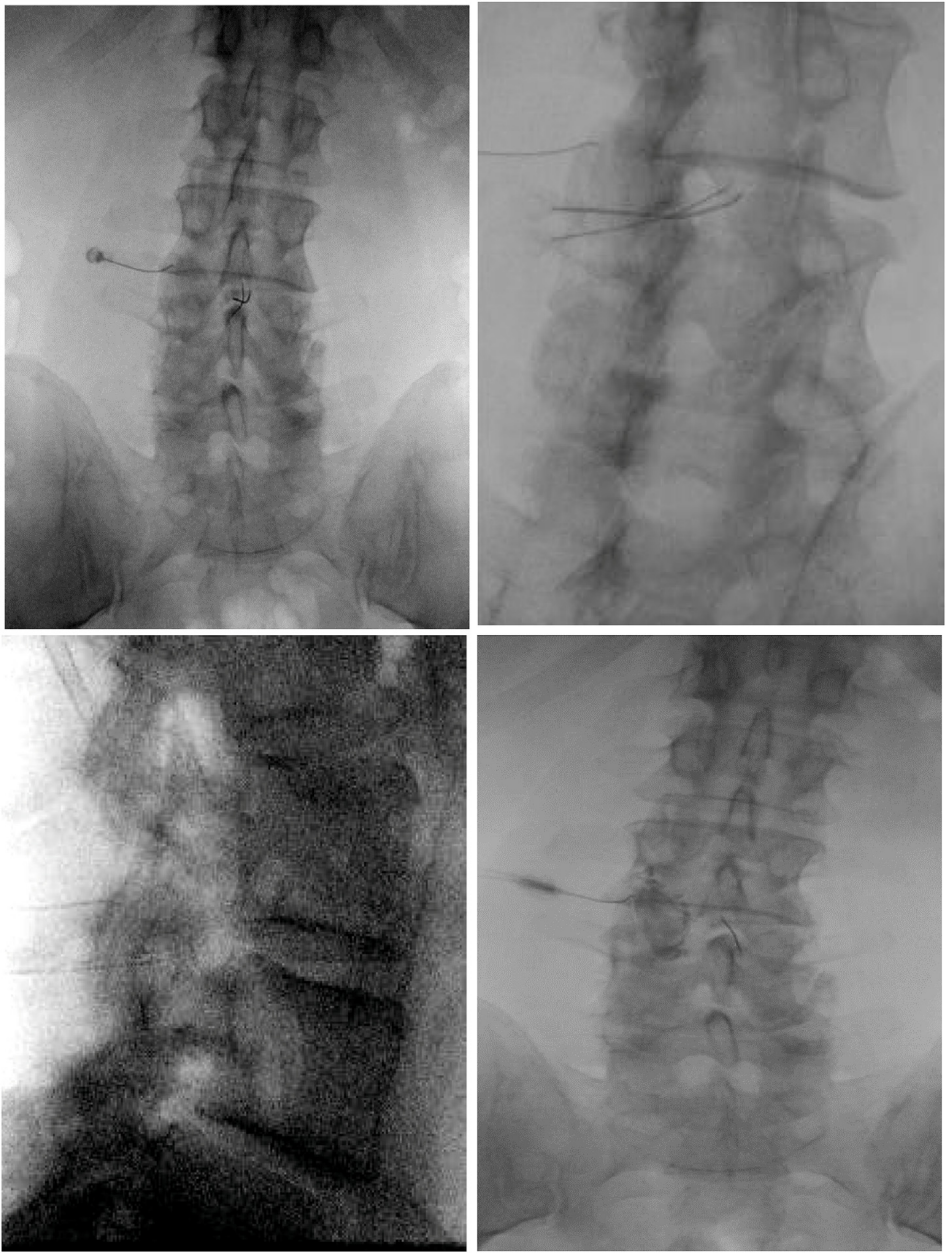
AP, oblique, and lateral intra-operative fluoroscopic images demonstrating one needle in the left L3-4 facet joint with subsequent arthrogram, and 2 needles presumed to be in the juxtafacet cyst.

Within two days of the procedure, the patient experienced a dramatic improvement in pain and leg strength and subsequently elected to cancel his scheduled laminoforaminotomy. Over the next three weeks, he progressively regained strength of his right leg and had complete resolution of pain. Although he exhibited slight residual weakness (5-/5) in right toe extension and ankle dorsiflexion, all other muscle groups had regained full strength and his gait normalized.

In the case of our patient, we did not directly observe evidence of cyst aspiration, and post-procedure radiographic confirmation of cyst shrinkage was not obtained. However, the patient's weakness, which had been progressively worsening, rapidly improved after the procedure, suggesting that cyst drainage was successfully accomplished. While it is possible that the intra-articular facet joint steroid injection was responsible for some or even all of the relief in the patient's pain, we do not believe that the steroid injection was responsible for the dramatic improvement in the patient's motor deficit. We are unaware of any literature to support that this would occur from an intra-articular steroid injection. Additionally, the patient's pain was radicular in nature, not axial low back pain, so we suspect that the relief in his pain was more likely a result of the cyst puncture/drainage and not from the intra-articular steroid injection. The patient was contacted one year after the procedure, and he reported no low back pain, no leg pain, and normal strength in his leg. Additionally, review of his chart 4 years later demonstrated that he continued to be an active patient in our electronic medical record system, however no additional appointments or diagnostic studies were done for the lumbar spine, and he did not undergo a lumbar spine operation or any additional invasive lumbar spine procedures.

This case suggests that percutaneous cyst puncture/aspiration is reasonable to consider as a first-line treatment for symptomatic facet joint cysts, even those with motor weakness, and may allow for avoidance of surgical intervention.

## Declaration of competing interest

The authors report no relevant disclosures or conflicts of interest, and no funding sources.
